# Free versus Fixed Combination Antihypertensive Therapy for Essential Arterial Hypertension: A Systematic Review and Meta-Analysis

**DOI:** 10.1371/journal.pone.0161285

**Published:** 2016-08-22

**Authors:** Samir G. Mallat, Bassem Y. Tanios, Houssam S. Itani, Tamara Lotfi, Elie A. Akl

**Affiliations:** 1 Department of Internal Medicine, Division of Nephrology, American University of Beirut, Beirut, Lebanon; 2 Department of Internal Medicine, Division of Nephrology, Makassed General Hospital, Beirut, Lebanon; 3 Clinical Research Institute, American University of Beirut, Beirut, Lebanon; 4 Department of Internal Medicine, American University of Beirut, Beirut, Lebanon; Cardiff University, UNITED KINGDOM

## Abstract

**Background:**

In a free drug combination, each Blood pressure (BP)-lowering drug is administered as a separate pill, while in a fixed drug combination several BP-lowering agents are combined in a single pill. Using a single pill may enhance compliance and simplify treatment, which would translate into better clinical outcomes. The objective of this meta-analysis is to compare the effects of using a fixed combination versus free combination of BP-lowering agents in the management of patients with essential hypertension.

**Methods:**

We searched Cochrane CENTRAL, MEDLINE, and EMBASE for randomized clinical trials (RCTs) addressing the objective of the review and assessing at least one of the following outcomes: BP-lowering efficacy, rapidity in achieving BP target, compliance, incidence of side effects, mortality, and morbidity. Two review authors independently selected eligible studies, abstracted data, and assessed risk of bias of included trials. The primary meta-analyses used a random-effects model.

**Results:**

We identified seven RCTs with a total of 397 participants. Meta-analysis of efficacy in controlling BP showed a non-significant reduction of mean systolic BP of 0.81 mmHg (95% CI -3.25, 1.64) favoring the fixed combination group. As for adverse events, results showed a non-significant 13% risk reduction favoring the free combination (risk ratio 1.13, 95% CI 0.85, 1.5). Low quality of evidence was noted for both outcomes. Rapidity in achieving BP target was assessed in only one trial, and the results favored the fixed combination. Adherence to treatment was assessed in three trials, no pooled analysis was possible for this outcome. None of the included trials assessed mortality and morbidity.

**Conclusion:**

The available low quality evidence does not confirm or rule out a substantive difference between fixed combination and free combination therapy in the management of HTN. Well designed RCTs with a long duration of follow-up and assessment of morbidity and mortality outcomes are needed.

## Introduction

Arterial Hypertension (HTN) is a highly prevalent disease, with estimates reaching 26% of the worldwide adult population.[1] In the United States, the prevalence of HTN reached 30%, as defined by a systolic blood pressure (BP) of 140 mmHg or higher, a diastolic BP of 90 mmHg or higher, or currently using BP-lowering drugs.[2] HTN remains one of the major preventable risk factors for coronary events, cerebro-vascular disease, heart failure, peripheral vascular disease and progression of kidney disease.[3–5] Most patients with HTN will require more than one drug to achieve BP target, and monotherapy would only be sufficient in about 20–30% of patients.[6] In addition, around 24% to 32% of patients will require a combination of more than two drugs to achieve BP targets.[7,8]

In a recent meta-analysis, a target systolic BP of less than 130 mmHg significantly decreased the incidence of cardiovascular events, [9] and in the recently published SPRINT trial, a mean number of BP medications of 2.8 was required to achieve a mean systolic BP of 121.5 mmHg in the intensive treatment group, which resulted in a 25% lower relative risk of cardiovascular events as compared to the standard-treatment group.[10]

Combination therapy for HTN may be delivered either as free or fixed drug combinations. In a free drug combination, each BP-lowering drug is administered in a separate pill, while in a fixed drug combination two or more agents are combined in a single pill (SPC). SPCs may offer several advantages over free drug combinations, such as better compliance and simplicity of treatment. The recently updated European guidelines have advocated SPCs as the preferred approach to combine BP-lowering drugs.[11]

As a result of the decreased pill burden, SPCs may increase adherence with the prescribed regimen. This would likely lead to increased overall BP-lowering efficacy, which would translate into decreased incidence of cardiovascular morbidity and mortality.[12] In addition, the use of SPCs may simplify the task of adjusting and titrating the doses of the component agents. This would translate into more rapid achievement of BP target which has been shown to correlate with better clinical outcomes.[13]

Any intervention that would help increase BP-lowering efficacy, decrease therapy side effects, and help increase compliance and adherence will likely have a major impact on decreasing cardiovascular morbidity and mortality.[14] A previous systematic review that included both retrospective and prospective clinical studies found that SPCs were associated with a 29% increase in compliance as compared to corresponding free-drug combination. However, the results of the analysis were inconclusive concerning BP-lowering efficacy and side effects.[15] Another systematic review included 12 retrospective observational studies, and found that the use of SPCs was associated with better medication adherence and lower health-care cost as compared to their free-drug counterparts.[16] Since the publication of these two systematic reviews, at least one potentially eligible randomized clinical trial has been published.[17] Furthermore, there is a need to summarize and evaluate evidence from studies having the least risk of bias, i.e., randomized trials.

The objective of this systematic review is to compare the beneficial and harmful effects of a fixed versus free combination of two or more BP-lowering agents in patients with essential HTN. Our systematic review, and as compared to other previously published systematic reviews on the subject, that included both randomized and non-randomized studies, will seek to include only randomized clinical trials, which would provide the highest degree of evidence.

## Material and Methods

### Protocol and registration

Our review is registered in PROSPERO, PROSPERO 2015:CRD42015026500 Available from http://www.crd.york.ac.uk/PROSPERO/display_record.asp?ID=CRD42015026500.

### Eligibility criteria

Only randomized clinical trials were considered to be included in the present systematic review. Inclusion criteria were patients with essential HTN, age greater than 18 years old from any ethnic or racial background. Exclusion criterion was patients with secondary HTN. The included studies would have an intervention arm consisting of a fixed drug combination, including two or more BP-lowering agent, and a control arm consisting of a free drug combination of the corresponding, equivalent dose components, given separately as two or more pills.

All co-interventions (including additional BP medications) should be similar for the two groups at study entry.

The pre-specified primary outcomes were, efficacy in controlling BP as determined by the systolic BP at the conclusion of the study, and rapidity in achieving BP target as defined by individual included trials. Secondary outcomes included adherence to treatment, adverse events, mortality, and morbidity (including cardiovascular outcomes such as coronary events, stroke, progression of peripheral vascular disease and kidney disease)

### Search strategy

We searched the following sources from inception to May 2015: the Cochrane Central Register of Controlled Trials (Central), MEDLINE, and EMBASE.

Details of the electronic search strategies are presented in S1 Table.

We did not use any language restrictions.

We also searched trials registries (www.controlled-trials.com/, www.clinicaltrialsregister.eu/, http://apps.who.int/trialsearch/).

For every included study we searched for any related protocol published either in databases of ongoing trials or in a peer reviewed journal.

We also searched the reference lists of included trials, related systematic reviews, and related health-technology assessment reports.

We contacted experts in the field to identify any additional potentially eligible studies to be included in our analysis.

### Selection process

Two review authors (BT, HI) screened in duplicate and independently the abstract and title of every record retrieved by the searches for potential eligibility. We retrieved the full text for all articles judged as potentially eligible by at least one of the two reviewers.

The two reviewers assessed in duplicate and independently the full texts for eligibility using a standardized and pilot tested screening form. They then compared their results and resolved any disagreements by consensus and, when unsuccessful, with the help of a third reviewer (SM). Before starting the selection process, BT and HI conducted calibration exercises to ensure the validity of the selection process.

We will present a PRISMA (preferred reporting items for systematic reviews and meta-analyses) flow-chart to summarize the study selection process.[18]

### Data extraction

For studies that fulfilled eligibility criteria, two review authors (BT, HI) independently and in duplicate abstracted relevant information using standardized data extraction forms. They then compared their results and resolved any disagreements by discussion and, when unsuccessful, with the help of a third reviewer (SM). We extracted information about the study design, the characteristics of the population, intervention, comparator, and outcomes.

### Assessment of risk of bias

Two review authors (BT, HI) assessed the risk of bias of each included study independently and in duplicate. We resolved disagreements by consensus and, when unsuccessful, with the help of a third reviewer (SM). Risk of bias was assessed using The Cochrane Collaboration's Risk of Bias tool.[19, 20] The following criteria were used: random sequence generation (selection bias); allocation concealment (selection bias); Blinding of participants, providers, data collectors, outcome adjudicators, and data analysts (performance bias and detection bias); Incomplete outcome data (attrition bias); Selective outcome reporting (reporting bias).

We judged risk of bias criteria as 'low risk', 'high risk' or 'unclear risk' and evaluated individual bias items as described in the Cochrane Handbook for Systematic Reviews of Interventions.[19]

### Data analysis

We expressed dichotomous data as risk ratio (RR) or hazard ratio (HR) with 95% confidence intervals (CI). For continuous outcomes data, we calculated the mean difference with 95% CI when trials used the same scale and the standardized mean difference when trials used different scales.

We attempted to obtain relevant missing data from authors. Three of the included trials, did not report standard deviations in the assessment of the primary outcome mean systolic BP. [17, 21, 22] We tried to contact the authors for any unpublished missing data that could assist us in calculating the missing standard deviations values, however we were not able to obtain this data. To account for the missing standard deviation we used the median standard deviation from the three included trials that reported mean systolic BP and standard deviations as described elsewhere.[23]

In terms of evidence synthesis, we used a random-effects model for the primary meta-analysis.[24] We assessed heterogeneity (inconsistency) between study results by visual inspection of the forest plots and by using the I^2^ statistic. We considered an I^2^ statistic of 50% or more as indicative of a considerable level of heterogeneity.[20] In order to explain any heterogeneity, we planned to conduct subgroup analyses based on the following categories: patients with advanced HTN (e.g., Stage II defined as systolic BP ≥160 or diastolic BP ≥ 100) versus early HTN, specific drug combinations, older age (e.g., more than 65) versus younger age.

We planned to perform sensitivity analyses in order to explore the influence on pooled effect sizes of the following: restricting the analysis to studies with low risk of bias; and restricting the analysis to large studies and studies with longer follow-up.

### Grading of the certainty of evidence

We graded the quality of the evidence for each outcome using the Grading of Recommendations Assessment, Development and Evaluation (GRADE) approach. The approach classifies the quality of evidence in into four categories: high, moderate, low and very low. It takes into account the following factors: risk of bias, imprecision, inconsistency, indirectness, and publication bias.[25]

We developed a Summary of Findings table using the GRADEpro/GDT tool.[26]

## Results

### Search Results

**Fig 1:**
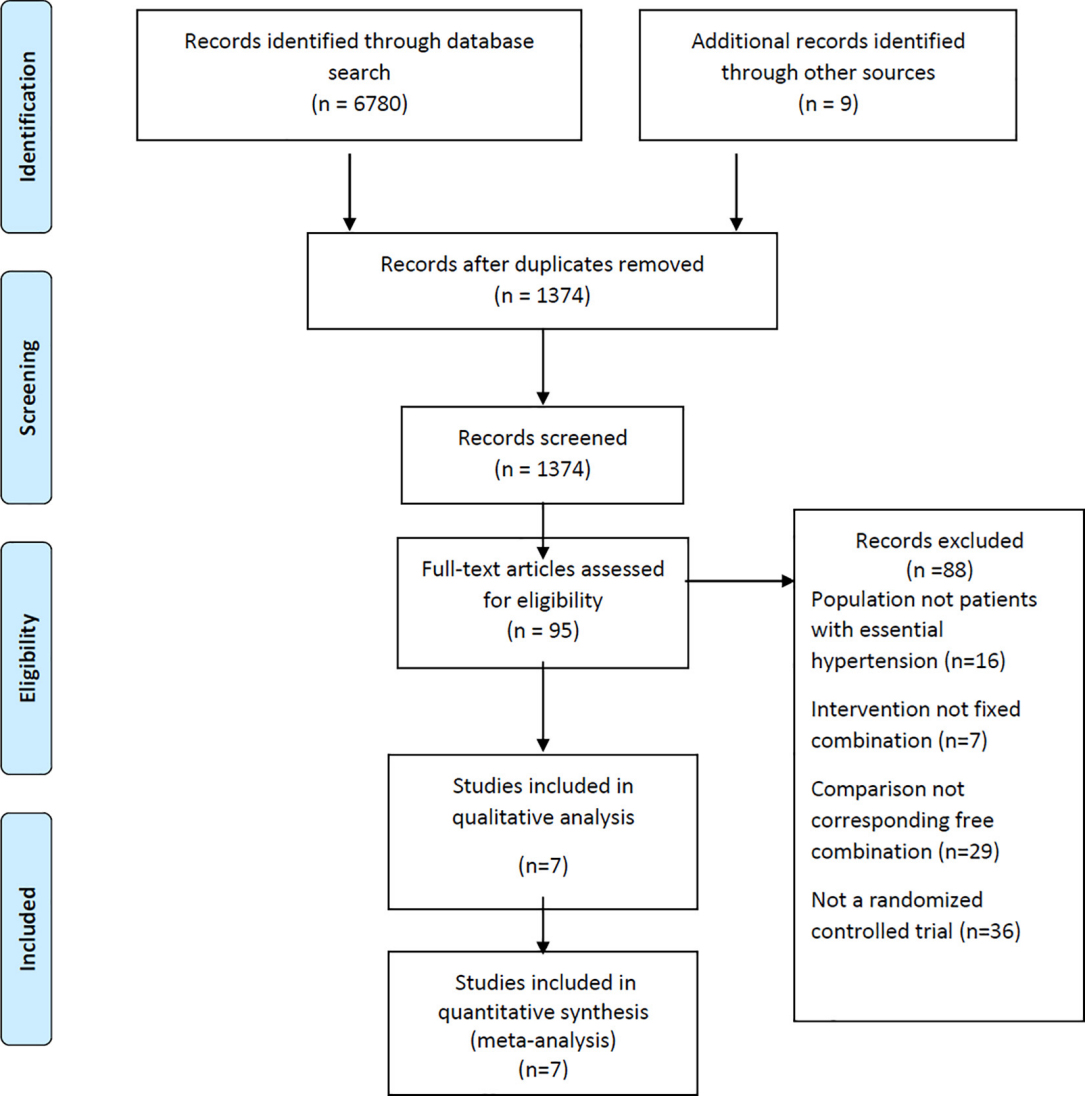
Free vs. Fixed combination antihypertensive therapy for essential arterial hypertension: PRISMA Flow chart.

Fig 1 shows the study flow chart. Out of 1374 screened citations, we identified seven eligible studies with a total of 397 participants.

### Included studies

Table 1 summarizes the characteristics of the seven included studies. Six trials were in the English language, and 1 trial was in Russian. All studies were randomized. One trial used a parallel group design, [17] one used a randomized, controlled, within-patient study design, [27] and the remaining 5 trials used a cross-over design.[21, 22, 28–30]. Source of funding for all the included studies was not specified.

**Table 1:** Characteristics of included studies.

Study Name	Study Design	Participants	Intervention	Control	Outcomes assessed
Jaattela 1979 [21]	Randomized, double-blind, crossover study	• Country: Finland • N = 21 • Mean age:52 • Sex: 38% men, 62% women • Initial mean lying BP: 180/110 mmHg	Propranolol 80 mg + Bendrofluazide 2.5 mg given as fixed combination pill twice daily	Propranolol 80 mg + Bendrofluazide 2.5 mg given as free combination twice daily	• Sitting and lying BP after 4 weeks • Adverse events
Nissinen 1980 [27]	Randomized, controlled, double-blind, within patient, crossover study	• Country: Finland • N = 23 • Mean age: 48 • Sex: 65% men, 35% women • Initial BP: 156.2±4/102.5±1.7 mm Hg	Atenolol 100 mg + Chlorthalidone 25mg in 1 fixed combination tablet	Atenolol 100mg, 1 tablet + Chlorthalidone 25mg, 1 tablet, given as free combination	Office BP measurement at 2 and 4 weeks
Solomon 1980 [22]	Randomized, double-blind, within patient, crossover study	• Country: England • N = 14 • Mean age: 44 • Sex: 50% men, 50% women • Mean BP at study entry: 178/116 mmHg	Propranolol 80mg + bendrofluazide 2.5mg given as a single tablet (inderetic), twice daily	Propranolol 80mg + bendrofluazide 2.5mg given separately twice daily	• Office BP at 4 weeks • Adverse events
Asplund 1984 [28]	Multicenter randomized, crossover study	• Country: Sweden • N = 160 • Mean age: 51 • Sex: 61% men, 39% women • Mean Initial BP: 146±16/92±8 mm Hg	Pindolol 10 mg + clopamide 5 mg as a combination tablet once daily	Pindolol 10 mg + clopamide 5 mg as 2 separate tablets once daily	• Office BP at 4 month • Heart rate • Compliance • Patient preference
Olvera 1991 [29]	Randomized, prospective, open label, crossover study	• Country: Mexico • N = 29 • Age between 30 and 70 years • Initial Mean BP in the free combination group 168±9/104±5 mm Hg • Initial Mean BP in the fixed combination group 162±21/104±6 mm Hg	Lisinopril 20 mg + HCTZ 12.5 mg in a single daily tablet	Lisinopril 20 mg + HCTZ 12.5 mg in separate tablets	• Office BP at 6 and 12 weeks • Heart rate • Weight • Adverse events
McLay 2000 [30]	Double-blind, placebo controlled, randomised, three way crossover multicenter study	• Country: United Kingdom • N = 26 • Mean age: 69 • Sex: 57% men, 43% women • Race: Caucasian • Mean sitting BP at randomization 172 ±15/102 ±6 mmHg	Felodipine ER/Metoprolol CR/ZOC 50mg, fixed combination	Felodipine ER/Metoprolol CR/ZOC 50mg, free combination	• 26 hours ambulatory BP monitoring after 12 weeks • Compliance • Adverse events
Pecherina 2014 [17]	Randomized, prospective, parallel groups study	• Country: Russia • N = 124 • Intervention group: 61% men, 39% women, age: 56 • Control group: 56.5% men, 43.5% women, age: 54	Nebivolol + Amlodipine as fixed combination (Nebilong AM 2.5/2.5 mg or 5/5mg)	Nebivolol 2.5 mg or 5mg in free combination with Amlodipine 2.5mg or 5mg	• 24 hours ambulatory BP monitoring at 3 months • Heart rate • Compliance • Quality of life

In four of the included trials, a combination of a beta-blocker and a diuretic was used, in one trial a combination of a renin angiotensin system (RAS) blocker and a diuretic was used, and in 2 trials a combination of a calcium channel blocker and a beta-blocker was used.

### Risk of bias in included studies

**Fig 2:**
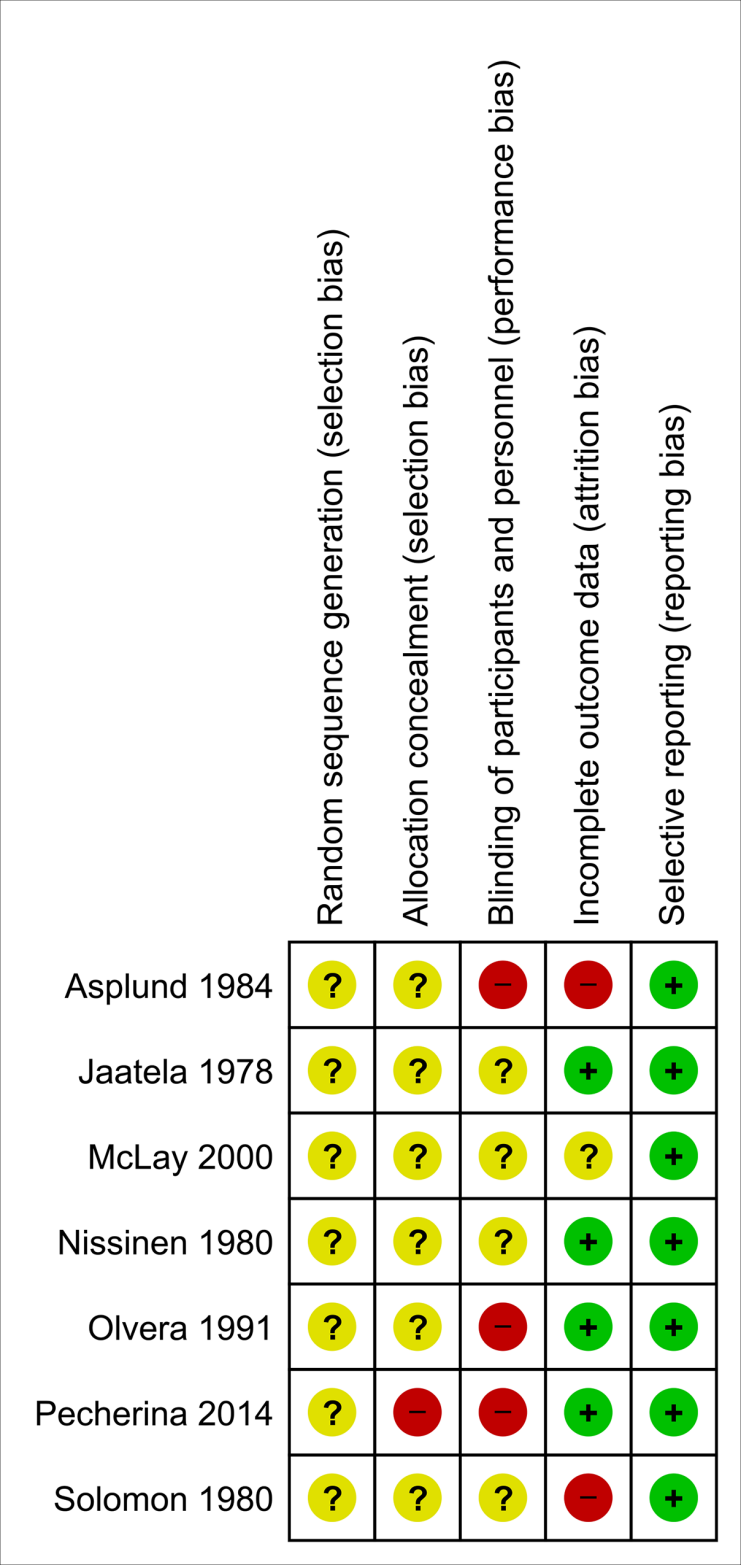
Risk of bias summary: review authors' judgements about each risk of bias item for each included study. (+): low risk of bias, (-): high risk of bias, (?): unclear risk of bias.

Table 2 and Fig 2 summarize the assessment of risk of bias in included studies.

**Table 2:** Risk of bias in included studies.

Study Name	Random sequence generation	Allocation concealment	Blinding	Completeness of data	Selective outcome reporting
Jaattela 1979 [21]	Unclear risk of bias; Method of randomization not specified.	Unclear risk of bias; Method of allocation concealment not specified.	Unclear risk of bias; Method of blinding not specified	Low risk of bias; “No patient was withdrawn from any treatment group”	Low risk of bias; All outcomes listed in the methods section are reported in the results section
Nissinen 1980 [27]	Risk of bias unclear; Method of randomization not specified.	Risk of bias unclear; Method of allocation concealment not specified.	Risk of bias unclear; Method of blinding not specified	Low risk of bias; “No patient was withdrawn from the study”	Low risk of bias; All outcomes listed in the methods section are reported in the results section
Solomon 1980 [22]	Risk of bias unclear; Method of randomization not specified	Risk of bias unclear; Method of allocation concealment not specified.	Risk of bias unclear; Method of blinding not specified	High risk of bias; “1 patient was withdrawn because of general feeling of fatigue, 5 other patients were withdrawn from the study because of non-attendance”	Low risk of bias; All outcomes listed in the methods section are reported in the results section
Asplund 1984 [28]	Risk of bias unclear; Method of randomization not specified	Risk of bias unclear; Method of allocation concealment not specified.	High risk of bias; “The patients were initially informed about the antihypertensive drugs given”	High risk of bias; “30 patients discontinued the study”	Low risk of bias; All outcomes listed in the methods section are reported in the results section
Olvera 1991[29]	Risk of bias unclear; Method of randomization not specified	Risk of bias unclear; Method of allocation concealment not specified.	High risk of bias; “open label study”	Low risk of bias; “One patient in control group dropped out due to severe cough”	Low risk of bias; All outcomes listed in the methods section are reported in the results section
McLay 2000[30]	Risk of bias unclear; Method of randomization not specified	Risk of bias unclear; Method of allocation concealment not specified.	Risk of bias unclear; Method of blinding not specified	Risk of bias unclear; “26 patients were randomized, 23 patients completed the study”	Low risk of bias; All outcomes listed in the methods section are reported in the results section
Pecherina 2014 [17]	Risk of bias unclear; Method of randomization not specified	Risk of bias unclear; Method of allocation concealment not specified.	High risk of bias; Open label study	Low risk of bias; All patients completed the study	Low risk of bias; All outcomes listed in the methods section are reported in the results section

In terms of random sequence generation, none of the included trials reported on the method used, none of the included trials reported on the method of allocation concealment. In three of the included trials, blinding was not clear, three of the included trials were open label, and therefore not blinded. Completeness of data was adequate in four of the included trials. In one trial (Asplund 1984), 30 patients discontinued the trial with unclear information regarding the distribution of drop-outs between the intervention and control groups, and in another trial (Solomon 1980), 6 out of 20 patients did not complete the trial, therefore the risk of bias in both of these trials was judged as high.[22, 28] In the third trial (McLay 2000), 3 out of 26 randomized patients did not complete the trial, and the risk of bias was judged as unclear.[30] In terms of selective outcome reporting, low risk of bias was judged for all included trials.

### Effects of intervention

#### Mean Systolic Blood pressure

**Fig 3:**
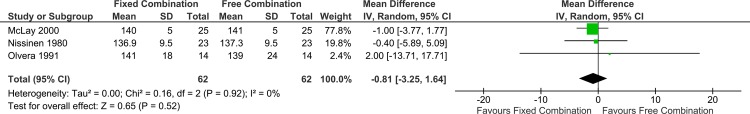
Forest plot for the effect of fixed vs free antihypertensive drug therapy on mean systolic blood pressure.

Fig 3 represents the forest plot of the comparison of fixed versus free antihypertensive therapy for the outcome of mean systolic blood pressure.

The main analysis included three trials, [27, 29, 30] but excluded four trials for the following reasons: three trials (Jaatela 1978, Solomon 1980, and Pecherina 2014) [17, 21, 22] did not report standard deviation, while the fourth trial (Asplund 1984)[28] reported mean change in systolic BP.

The meta-analysis showed a non-significant mean reduction of systolic blood pressure of 0.81 mmHg (95% CI -3.25, 1.64), with the direction favoring the fixed combination group. There was no heterogeneity (I² = 0%). Quality of the evidence was deemed to be low, due to high risk of bias and imprecision.

**Fig 4:**
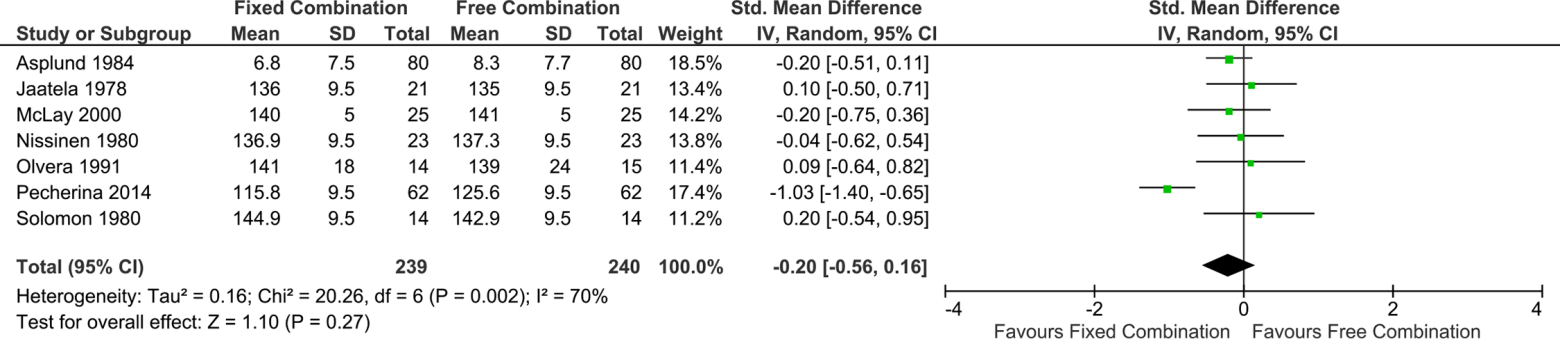
Forest plot for the effect of fixed vs. free antihypertensive drug therapy on mean systolic blood pressure, using standardized mean difference.

In the sensitivity analysis, including all seven studies, after imputing the missing standard deviations, the results did not change. As described above, we imputed the standard deviations by using the value 9.5mmHg, the median of the standard deviations reported by the three trials included in the main analysis. Also, we calculated the standardized mean difference to incorporate the trial that reported mean change in systolic BP and not mean systolic BP (Asplund 1984).[28] While the results remained with no statistical significance, heterogeneity was significant (I² = 69%), (Fig 4).

#### Adverse events

**Fig 5:**
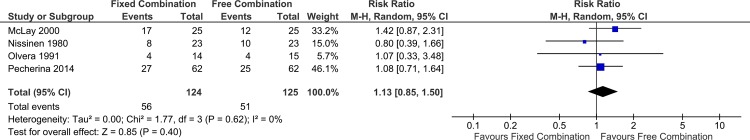
Forest plot for the effect of fixed vs. free antihypertensive drug therapy on adverse Events.

Fig 5 represents the forest plot for the effects of fixed versus free antihypertensive therapy on adverse events.

All of the included studies expect two (Jaattela 1979, Solomon 1980)[21,22] reported incidence of adverse events, and were included in the pooled analysis. The results showed a nonsignificant 13% risk reduction in adverse events favoring the free combination group (RR 1.13, 95% CI 0.85, 1.5). Quality of evidence regarding this outcome was deemed to be low due to the high risk of bias and imprecision.

#### Patients with controlled Blood pressure

**Fig 6:**
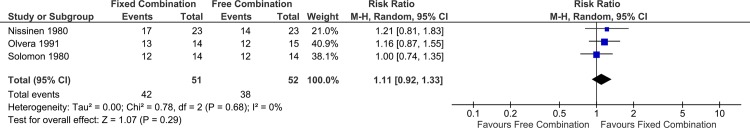
Forest plot for the effect of fixed versus free antihypertensive drug therapy on the control of blood pressure.

Fig 6 represents the forest plot for the effect of fixed versus free antihypertensive therapy on control of BP. We included in this analysis three trials (Nissinen 1980, Solomon 1980, Olvera 1991)[22, 27, 29] that reported on the numbers of patients achieving BP target at the conclusion of the trial. We excluded one trial (Pecherina 2014)[17], that did not report on the exact numbers. In that trial, the authors implied a possible advantage of the fixed combination regarding this outcome, since addition of a thiazide diuretic was necessary to achieve BP target in 1.6% of patients in the intervention group versus 2.3% in the control group. The results of the pooled analysis of the three trials showed a nonsignificant trend towards better blood pressure control (risk ratio 1.11 95% CI 0.92, 1.33) favoring the fixed combination group. The quality of the evidence was deemed to be low due on the high risk of bias and imprecision.

#### Adherence to treatment

Three of the included trials reported on adherence and compliance to treatment. No pooled analysis was possible for this outcome because of either missing data or differences in the methods of outcome measurement. Both Asplund 1984, and McLay 2000 reported no difference between the 2 groups with regards to compliance.[28, 30] The third trial (Pecherina 2014) showed increased compliance in the fixed combination group.[17]

#### Other outcomes

Only one trial (Pecherina 2014)[17] reported on the rapidity in achieving BP target, and the results showed that patients in the fixed combination group had significantly lower values of systolic and diastolic BP starting week 2, as compared with patients in the free combination group.

All of the included trials were short term and none of them reported on mortality or cardiovascular morbidity outcomes.

#### Subgroup analysis, investigation for heterogeneity, and sensitivity analysis

In our analysis of outcomes, little heterogeneity was detected, and it was not possible to conduct the pre-planned subgroup analysis due to the small number of included studies.

## Discussion

In summary, we found low quality evidence that does not confirm or rule out a substantive difference in benefits or harms between fixed combination and free combination of antihypertensive regimen in the management of patients with HTN. (Table 3).

**Table 3:** Summary of findings table: Fixed antihypertensive drug therapy compared to free antihypertensive drug therapy for essential arterial hypertension.

Outcomes	№ of participants (studies)	Quality of the evidence (GRADE)^1^	Relative effect (95% CI)	Anticipated absolute effects	
				Risk with Free antihypertensive drug therapy	Risk difference with Fixed antihypertensive drug therapy^2^
Mean systolic blood pressure	124 (3 RCTs)	⨁⨁◯◯ LOW^3^^,^^4^	-	The mean systolic blood pressure was **139.1** mmHg	MD **0.81 mmHg lower** (3.25 lower to 1.64 higher)
Adverse Events	249 (4 RCTs)	⨁⨁◯◯ LOW^3^^,^^4^	**RR 1.13** (0.85 to 1.50)	**Study population**	
				408 per 1000	**53 more per 1000** (61 fewer to 204 more)
Patients with controlled Blood pressure	103 (3 RCTs)	⨁⨁◯◯ LOW^3^^,^^4^	**RR 1.11** (0.92 to 1.33)	**Study population**	
				731 per 1000	**80 more per 1000** (58 fewer to 241 more)

**CI:** Confidence interval; **MD:** Mean difference; **RR:** Risk ratio

^1^**GRADE Working Group grades of evidence:**

**High quality:** We are very confident that the true effect lies close to that of the estimate of the effect

**Moderate quality:** We are moderately confident in the effect estimate: The true effect is likely to be close to the estimate of the effect, but there is a possibility that it is substantially different

**Low quality:** Our confidence in the effect estimate is limited: The true effect may be substantially different from the estimate of the effect

**Very low quality:** We have very little confidence in the effect estimate: The true effect is likely to be substantially different from the estimate of effect

^2^**The risk in the intervention group** (and its 95% confidence interval) is based on the assumed risk in the comparison group and the **relative effect** of the intervention (and its 95% CI).

^3^Unclear or high risk of bias in included trials

^4^Confidence interval does not rule out or confirm difference between the intervention and control groups

### Strengths and limitations

One strength of this systematic review is inclusion of only prospective, randomized clinical trials, which are at lower risk of bias relative to non-randomized trials and observational studies. Previously published systematic reviews on the topic included retrospective studies. [16] Also we excluded studies which did not use the same drug regimens (i.e., used different drugs and/or different doses) in the free combination and fixed combination groups, and which previous systematic reviews included.[15, 31–35].

Limitations of this systematic review relate to those of the existing literature. These include the relatively low number of published trials, the limited number of participants, the overall low quality of evidence, the relatively short follow-up, and the lack of assessment of important patient outcomes. Furthermore, with regards to the risk of bias assessment, we used the “unclear” category option when it was not explicit whether the specific methodological feature was met or not. This reflects the poor reporting of the methodological features of the included trials. In addition four trials did not report standard deviations (SDs) in the reporting of their outcomes, so we had to substitute the missing SDs with the median SD of the three other included trials. Also, four of the seven included trials used a combination of a beta-blocker and a diuretic, a combination which is not considered a preferred combination in the modern management of HTN. [36].These findings clearly identify the current gaps in the existing literature relating to this major topic.

### Comparison to other systematic reviews

Results of previously published systematic reviews on the subject favored the use of fixed combination therapy in the management of HTN. In an analysis of 15 retrospective studies, Sherrill et Al. demonstrated increased adherence and persistence to therapy with subsequent reduced healthcare costs with the use of a fixed combination regimen. [16]. In another systematic review Gupta et Al. demonstrated a significant improvement in compliance, and nonsignificant trends in BP control and adverse events favoring the use of a fixed combination. [15]. Our review differs from these systematic reviews in few aspects. First, we strictly included randomized clinical trials. Second, we used rigorous methodology for assessing the included trials for risk of bias, i.e., the Cochrane Collaboration Risk of Bias tool. Third, we assessed the quality of evidence by outcome using the GRADE methodology and constructed a Summary of Findings table (SoF) summarizing the statistical data as well as the quality of evidence by outcomes of interest.

As stated above, the results of these previously published systematic reviews showed a significant trend towards increased compliance to therapy favoring the fixed combination therapy, however these results were largely based on retrospective data. [15, 16]. Furthermore, these systematic reviews were inconclusive regarding BP efficacy and incidence of side effects. In fact, the meta-analysis by Gupta et Al. revealed a statistically nonsignificant reduction of 4.1 mmHg (95% CI:–9.8 to 1.5 mm Hg; P = 0.15) in systolic and 3.1 mm Hg (95% CI: -7.1 to 0.9 mm Hg; P = 0.13) in diastolic BP, favoring the fixed combination group. Likewise, the analysis showed a 20% decrease in adverse events favoring the fixed combination group, that did not reach statistical significance (OR: 0.80 [95% CI: 0.58 to 1.11]). [15].

In a recent nested matched case-control analysis, use of a fixed combination antihypertensive therapy was associated with an approximate 2-fold increased risk of serious adverse events, including hypotension, syncope, and collapse, leading to more hospitalizations, as compared to same components of therapy used as free combination. Occurrence of serious adverse events may impact negatively on compliance, as patients and physicians will be reluctant to resume these medications, which in turn, will have negative implications on long-term BP control and cardio-vascular outcomes.[37] This study and the results of our meta-analysis highlight the need for properly designed randomized controlled trials (RCTs) with head to head comparison of fixed versus free drug combination regimens with regards to BP-lowering efficacy and adverse events.

### Implications for practice

Several international guidelines, such as the European guidelines, advocated the use of fixed drug combination whenever possible, in an effort to improve adherence to therapy. This recommendation is given a class IIb evidence (Usefulness/efficacy is less well established by evidence/opinion), based on the Gupta et Al. meta-analysis [11]. Guidelines from the American society of Hypertension suggested the use of a fixed drug combination to simplify the treatment regimen.[38] On the other hand, the recently published JNC 8 guidelines suggested the use of either strategy to combine antihypertensive drugs.[39]. We do not feel that our results can challenge the current recommendations; however, our review clearly demonstrates the lack of high quality evidence from RCTs to support the superiority of a fixed over a free drug combination in the management of HTN.

### Implications for research

Our search of the trial registries (www.controlled-trials.com, www.clinicaltrialsregister.eu/, http://apps.who.int/trialsearch/), yielded one ongoing international multicenter trial comparing the efficacy and safety of the fixed combination of indapamide and amlodipine in a single-pill, to the same drugs given separately in patients with mild to moderate uncontrolled essential hypertension.[40] This ongoing trial will recruit 150 patients and will have a 12 weeks duration of follow-up. It will assess systolic and diastolic BP parameters in addition to normalization of BP after 12 weeks, however it will not assess compliance or morbidity and mortality outcomes. There is a need for additional high quality evidence, and trials with longer follow-up to better guide clinical practice.

## Conclusions

Based on our systematic review of the existing data from RCTs, the available low quality evidence does not confirm or rule out a significant difference between using a fixed drug combination versus a free drug combination, with respect to blood pressure control and incidence of adverse events, in the management of HTN. The included studies have not adequately assessed the effect on compliance and rapidity in achieving blood pressure targets, however the available evidence suggests a trend towards better compliance and a more rapid achievement of blood pressure targets. If these effects are later confirmed, they could translate into a great impact at reducing cardiovascular morbidity associated with HTN. However, due to overall high risk of bias and low quality of included trials, the results of our systematic review should be interpreted cautiously. The main contribution to the literature of our systematic review is that it identified the lack of high quality evidence to support the superiority of one approach to combination therapy over the other in the management of HTN. There is a need for additional RCTs, with long follow-up and assessment cardiovascular and mortality outcomes, to better guide clinical practice.

## Supporting Information

S1 Table Electronic Search Details. (PDF)

S2 Table PRISMA Checklist. (PDF)
