# Supplementary material for: Free versus Fixed Combination Antihypertensive Therapy for Essential Arterial Hypertension: A Systematic Review and Meta-Analysis
Source: PLoS One. 2016 Aug 22;11(8):e0161285. doi: 10.1371/journal.pone.0161285 (PMC4993355; doi:10.1371/journal.pone.0161285)
Supplement: S2 Table — (PDF) [file pone.0161285.s002.pdf]

Appendix1: Electornic Search Details

Appendix 1, Table 1: Medline search strategy

MEDLINE	
**# ▲**	**Searches**
1	exp ANTIHYPERTENSIVE AGENTS/
2	exp hypertension/
3	exp blood pressure/
4	(high or elevated or raised or increas*).mp.
5	3 and 4
6	hypertens*.tw.
7	((high or elevated or raised or increas*) adj2 (blood pressure or bloodpressure)).mp.
8	1 or 2 or 5 or 6 or 7
9	exp drug combinations/
10	((single or fixed or bitherap* or bi-therap* or therap* or drug* or medicat* or medicin* or pill* or (one adj pill) or onepill) adj3 combin*).mp.
11	((single or fixed or bitherap* or bi-therap*) adj3 (dose* or dosage* or preparation* or formulation* or mixture*)).mp.
12	FDC.ti,ab.
13	SPC.ti,ab.
14	all-in-one.ti,ab.

15	(polypill* or poly-pill*).tw.
16	((single adj pill*) or singlepill*).tw.
17	(co-formulat* or coformulat*).mp.
18	(multi-ingredient* or multiingredient*).mp. [mp=title, abstract, original title, name of substance word, subject heading word, keyword heading word, protocol supplementary concept, rare disease supplementary concept, unique identifier]
19	9 or 10 or 11 or 12 or 13 or 14 or 15 or 16 or 17 or 18
20	exp drug therapy combination/
21	((unfixed or un-fixed or separate* or free or dual or two) adj3 combin*).mp.
22	((separate* or dual or two or add*) adj3 (monotherap* or mono-therap* or pill* or drug* or medicin* or medicat* or dose* or dosage* or preparation* or formulation* or mixture*)).mp.
23	(multitherap* or multi-therap*).mp.
24	(multi-drug* or multidrug*).mp.
25	(poly-therap* or polytherap*).mp.
26	(multiple adj drug*).tw.
27	(co-administ* or coadminist*).mp.
28	20 or 21 or 22 or 23 or 24 or 25 or 26 or 27
29	randomized controlled trial.pt.
30	controlled clinical trial.pt.

31	random*.mp.
32	parallel.tw.
33	trial.tw.
34	groups.ab.
35	dt.fs.
36	(doubl* adj3 blind*).mp.
37	29 or 30 or 31 or 32 or 33 or 34 or 35 or 36
38	animals/ not (humans/ and animals/)
39	37 not 38
40	exp compliance/
41	complian*.mp.
42	exp patient satisfaction/
43	(patient* adj satisfaction).mp.
44	exp medication adherence/
45	((medication* or drug* or therap*) adj3 (complian* or adheren*)).mp.
46	((medication* or drug* or therap*) adj3 ((non adj adheren*) or nonadheren* or (non adj complian*) or noncomplian*)).mp.
47	(persisten* adj3 (medication* or drug* therap*)).mp.
48	exp drug toxicity/
49	(side adj effect*).mp.
50	(adverse adj effect*).mp.

51	(stay-on adj3 (therap* or drug* or medicat*)).mp.
52	(patient* adj3 ((drop adj out*) or dropout*)).mp.
53	((drug* or medication* or therap*) adj3 toxicit*).mp.
54	40 or 41 or 42 or 43 or 44 or 45 or 46 or 47 or 48 or 49 or 50 or 51 or 52 or 53
55	8 and 19 and 28 and 39
**56**	**54 and 55**

Appendix 1, Table 2: EMBASE search strategy:

**EMBASE**	
# ▲	**Searches**
1	exp ANTIHYPERTENSIVE AGENTS/
2	exp hypertension/
3	exp blood pressure/
4	(high or elevated or raised or increas*).mp.
5	3 and 4
6	hypertens*.tw.
7	((high or elevated or raised or increas*) adj2 (blood pressure or bloodpressure)).mp.
8	1 or 2 or 5 or 6 or 7
9	exp drug combinations/

10	((single or fixed or bitherap* or bi-therap* or therap* or drug* or medicat* or medicin* or pill* or (one adj pill) or onepill) adj3 combin*).mp.
11	((single or fixed or bitherap* or bi-therap*) adj3 (dose* or dosage* or preparation* or formulation* or mixture*)).mp.
12	FDC.ti,ab.
13	SPC.ti,ab.
14	all-in-one.ti,ab.
15	(polypill* or poly-pill*).tw.
16	((single adj pill*) or singlepill*).tw.
17	(co-formulat* or coformulat*).mp.
18	(multiingredient* or multi-ingredient*).mp.
19	9 or 10 or 11 or 12 or 13 or 14 or 15 or 16 or 17 or 18
20	exp drug therapy combination/
21	((unfixed or un-fixed or separate* or free or dual or two) adj3 combin*).mp.
22	((separate* or dual or two or add*) adj3 (monotherap* or mono-therap* or pill* or drug* or medicin* or medicat* or dose* or dosage* or preparation* or formulation* or mixture*)).mp.
23	(multitherap* or multi-therap*).mp.
24	(multi-drug* or multidrug*).mp.
25	(poly-therap* or polytherap*).mp.

26	(multiple adj drug*).tw.
27	(co-administ* or coadminist*).mp. [mp=title, abstract, subject headings, heading word, drug trade name, original title, device manufacturer, drug manufacturer, device trade name, keyword]
28	20 or 21 or 22 or 23 or 24 or 25 or 26 or 27
29	random*.mp.
30	factorial*.mp.
31	(crossover* or cross-over*).mp.
32	placebo*.mp.
33	((doubl* or singl*) adj blind*).mp. [mp=title, abstract, subject headings, heading word, drug trade name, original title, device manufacturer, drug manufacturer, device trade name, keyword]
34	(assign* or allocat* or volunteer*).mp. [mp=title, abstract, subject headings, heading word, drug trade name, original title, device manufacturer, drug manufacturer, device trade name, keyword]
35	Crossover Procedure/
36	Double-blind Procedure/
37	Randomized Controlled Trial/
38	Single-blind Procedure/
39	29 or 30 or 31 or 32 or 33 or 34 or 35 or 36 or 37 or 38
40	exp compliance/
41	complian*.mp.

42	exp patient satisfaction/
43	(patient* adj satisfaction).mp.
44	exp medication adherence/
45	((medication* or drug* or therap*) adj3 (complian* or adheren*)).mp.
46	((medication* or drug* or therap*) adj3 ((non adj adheren*) or nonadheren* or (non adj complian*) or noncomplian*)).mp.
47	(persisten* adj3 (medication* or drug* therap*)).mp.
48	exp drug toxicity/
49	(side adj effect*).mp.
50	(adverse adj effect*).mp.
51	(stay-on adj3 (therap* or drug* or medicat*)).mp.
52	(patient* adj3 ((drop adj out*) or dropout*)).mp.
53	((drug* or medication* or therap*) adj3 toxicit*).mp.
54	40 or 41 or 42 or 43 or 44 or 45 or 46 or 47 or 48 or 49 or 50 or 51 or 52 or 53
55	animals/ not (humans/ and animals/)
56	39 not 55
57	8 and 19 and 28 and 56
**58**	**54 and 57**

Appendix 1, Table 3: **Cochrane CENTRAL search Strategy**

**Cochrance Central**	
**#▲**	**Searches**
#1	MeSH descriptor: [Antihypertensive Agents] explode all trees
#2	MeSH descriptor: [Hypertension] explode all trees
#3	MeSH descriptor: [Blood Pressure] explode all trees
#4	high* or elevat* or increas* or rais*
#5	#3 and #4
#6	hypertens*
#7	#4 near/2 (bloodpressure or (blood near pressure))
#8	#1 or #2 or #5 or #6 or #7
#9	MeSH descriptor: [Drug Combinations] explode all trees
#10	((single* or fix* or bitherap* or bi-therap*) near/3 (dose* or dosage* or preparation* or formulation* or mixture*))
#11	((single* or fix* or bitherap* or bi-therap* or therap* or drug* or medicat* or medicin* or pill* or (one near pill) or onepill) near/3 combin*)
#12	FDC
#13	SPC
#14	all-in-one
#15	polypill* or poly-pill*

#16	single near pill* or singlepill*
#17	co-formulat* or coformulat*
#18	multiingredient* or multi-ingredient*
#19	#9 or #10 or #11 or #12 or #13 or #14 or #15 or #16 or #17 or #18
#20	((unfix* or un-fix or separate* or free or dual or two) near/3 combin*)
#21	((separate* or dual or two or add*) near/3 (monotherap* or mono-therap* or pill* or drug* or medicin* or medicat* or dose* or dosage* or preparation* or formulation* or mixture*))
#22	multitherap* or multi-therap*
#23	multi-drug* or multidrug*
#24	polytherap* or poly-therap*
#25	multiple near drug*
#26	co-administ* or coadminist*
#27	#20 or #21 or #22 or #23 or #24 or #25 or #26
#28	#8 and #19 and #27
#29	MeSH descriptor: [Patient Compliance] explode all trees
#30	MeSH descriptor: [Patient Satisfaction] explode all trees
#31	MeSH descriptor: [Medication Adherence] explode all trees
#32	((medication* or drug* or therap*) near/3 (complian* or adheren*))
#33	(medication* or drug* or therap*) near/3 ((non near adheren*) or nonadheren* or (non near complian*) or noncomplian*)

#34	persisten* near/3 (medication* or drug* or therap*)
#35	MeSH descriptor: [Drug Toxicity] explode all trees
#36	MeSH descriptor: [Patient Dropouts] explode all trees
#37	(side or adverse) near (effect* or reaction*)
#38	stay-on near/3 (therap* or drug* or medicat*)
#39	patient near (dropout* or drop-out*)
#40	(drug* or medicat* or therap*) near/3 toxicit*
#41	#29 or #30 or #31 or #32 or #33 or #34 or #35 or #36 or #37 or #38 or #39 or #40
#42	#28 and #41
